# Optical Coherence Tomography and Optical Coherence Tomography Angiography in Pediatric Retinal Diseases

**DOI:** 10.3390/diagnostics13081461

**Published:** 2023-04-18

**Authors:** Chung-Ting Wang, Yin-Hsi Chang, Gavin S. W. Tan, Shu Yen Lee, R. V. Paul Chan, Wei-Chi Wu, Andrew S. H. Tsai

**Affiliations:** 1 Department of Ophthalmology, Chang Gung Memorial Hospital, Linkou Medical Center, Taoyuan City 333, Taiwan; 2 Singapore National Eye Centre, Singapore, Singapore 168751, Singapore; 3 DUKE NUS Medical School, Singapore 169857, Singapore; 4 Department of Ophthalmology and Visual Sciences, University of Illinois at Chicago, Illinois Eye and Ear Infirmary, Chicago, IL 60612, USA; 5 College of Medicine, Chang Gung University, Taoyuan City 333, Taiwan

**Keywords:** optical coherence tomography, optical coherence tomography angiography, pediatric retina, retinopathy of prematurity

## Abstract

Indirect ophthalmoscopy and handheld retinal imaging are the most common and traditional modalities for the evaluation and documentation of the pediatric fundus, especially for pre-verbal children. Optical coherence tomography (OCT) allows for in vivo visualization that resembles histology, and optical coherence tomography angiography (OCTA) allows for non-invasive depth-resolved imaging of the retinal vasculature. Both OCT and OCTA were extensively used and studied in adults, but not in children. The advent of prototype handheld OCT and OCTA have allowed for detailed imaging in younger infants and even neonates in the neonatal care intensive unit with retinopathy of prematurity (ROP). In this review, we discuss the use of OCTA and OCTA in various pediatric retinal diseases, including ROP, familial exudative vitreoretinopathy (FEVR), Coats disease and other less common diseases. For example, handheld portable OCT was shown to detect subclinical macular edema and incomplete foveal development in ROP, as well as subretinal exudation and fibrosis in Coats disease. Some challenges in the pediatric age group include the lack of a normative database and the difficulty in image registration for longitudinal comparison. We believe that technological improvements in the use of OCT and OCTA will improve our understanding and care of pediatric retina patients in the future.

## 1. Introduction

The pediatric age group is diverse and ranges from the pre-verbal newborn to teenagers. A “One-size fits all” approach may not be applicable in the evaluation of pediatric retinal diseases. One of the most common reasons for fundus examination in a newborn is screening for retinopathy of prematurity (ROP) in the neonatal intensive care unit (NICU) [1]. ROP is traditionally assessed via indirect ophthalmology and a contact fundus imaging device, such as the Retcam (Natus Medical Systems, Inc., Pleasanton, CA, USA) with or without fluorescein angiography. Pediatric retinal disease is usually diagnosed, classified and staged based on a fundus examination or color fundus photography. Examination under general anesthesia is often required for detailed evaluation. An older child or teenager can be examined similarly to adults in a sitting position, although the ability to cooperate may be an issue.

The advent of optical coherence tomography (OCT) and OCT angiography (OCTA) revolutionized our understanding of retinal diseases [2]. OCT was first introduced by Huang et al. in 1991 [3] and it utilizes low-coherence reflectometry to image a two-dimensional cross-section of the retina that highly correlates with histology. OCT became indispensable for imaging various retinal diseases where structural changes in different layers of the retina can be visualized [3]. OCTA involves performing repeated B-scans. Coupled with the development of high-resolution spectral domain OCT (SD-OCT) and swept-source OCT (SS-OCT), OCTA enables depth-resolved visualization of the blood flow of different retinal capillary layers and segmented volumetric data without the need for dye-based angiography [4,5]. OCT and OCTA have been extensively utilized to examine adult retinal diseases, such as diabetic retinopathy and age-related macular degeneration.

Their use in the pediatric age group has been limited due to issues with cooperation and positioning. Some pediatric retinal specialists have improvised and successfully imaged the pediatric fundus in the “flying baby position”, both for widefield color fundus imaging and OCT [6,7]. To achieve supine imaging, commercially available handheld portable OCT devices, such as Bioptigen (Bioptigen Inc., Research Triangle Park, NC, USA), and investigational armature-based portable systems, such as the Heidelberg Spectralis Flex module (Heidelberg Engineering, Heidelberg, Germany), were developed [2]. However, these devices are not widely available.

In comparison to current imaging modalities, conventional wide-angle retinal image acquisition using Retcam (Natus Medical Systems, Inc., Pleasanton, CA, USA) requires contact with the ocular surface of infants and toddlers. By contrast, current handheld OCTs are non-contact. The development of other commercialized widefield image capture systems, including Optos (Optos PLC, Dunfermine, Scotland, UK) or the Heidelberg Spectralis ultra-widefield fundus imaging system, permitted visualization of the retinal periphery that does not require contact but needed more patient cooperation and had more limitations in terms of positioning. Therefore, they are only applicable to older children [8]. Fluorescein and indocyanine green angiography are invasive dye-based procedures that provide visualization of the retinal and choroidal vasculature respectively. They are able to detect non-perfusion and leakage in the retinal periphery, in contrast to OCTA. Yet, possible side effects from mild nausea to severe complications, such as anaphylactic shock, may limit a parent’s preference in opting for these procedures. Moreover, color fundus photography and angiography provide only *en face* images, in contrast to OCT and OCTA.

B scan ultrasonography is a non-invasive imaging modality that detects large retinal or choroidal lesions [9]. Compared with OCT, B scan ultrasonography has a much lower resolution and OCT is much more precise in detecting pre-retinal, intra-retinal and subretinal lesions.

To date, OCT and OCTA have been used to examine the pediatric retina to evaluate retinal development, pediatric retinal vascular diseases, inherited retinal degenerations, trauma, tumors, epiretinal membranes and inflammatory diseases [2,10,11]. Having cross-sectional information and quantitative vessel parameters of normal and diseased pediatric retinas may be helpful in the diagnosis, follow up and assessment of treatment response. The non-invasiveness of OCTA may also be particularly attractive in the pediatric age group.

A comprehensive review and update on the use of OCT and OCTA in pediatric retinas are needed. Therefore, in this article, we aimed to review the literature on the current research and clinical applications of OCT and OCTA in pediatric retinal diseases and their possible future development.

## 2. Methodology

A literature search was performed on Pubmed with the terms “optical coherence tomography” or “optical coherence tomography angiography” with “pediatric” and in combination with a term for a selected disease, including “retinopathy of prematurity”, “familial exudative vitreoretinopathy”, “Coats disease”, “incontinentia pigmenti”, “type I diabetes related retinopathy”, “inherited retinal disease” or “inherited retinal degeneration”, “neoplasm”, “uveitis” or “intraocular inflammation”, “nontraumatic brain injury” or “shaken baby syndrome”, and “torpedo maculopathy”. The selected articles were restricted to articles written in English. Several case series and reports were included if the author reported a robust finding of pediatric retinal disease using OCT or OCTA. The date range for the papers included in this review was from November 1991 to January 2023.

## 3. Normal OCT and OCTA in Children

When interpreting pediatric OCT, parameters including the macula, retinal nerve fiber layer thickness and optic nerve may be adjusted according to race, axial length and age. Different research groups proposed normative databases for quantifying parameters on OCT based on different ethnicities [12,13,14,15,16,17,18].

Modifications during imaging are necessary due to a shorter axial length (AL), steeper corneal curvature, greater astigmatism and refractive error. Age adjustments should also be made as the eye develops from that of a neonate to an infant and child [19,20]. When performing OCT on an infant, adjustment of the OCT scanning pivot position anterior to the iris plane (unlike in the iris plane in adults), shortening the OCT reference arm position and adjusting focus in consideration of refractive error (generally hyperopes) are needed [20]. A study looked at 6–9-year-old children and found that ocular magnification was more prominent in hyperopes, and choroidal thickness should be corrected in the nasal perifoveal region of individuals with ALs shorter than 22.8 mm or longer than 26.0 mm [21].

From infancy to childhood, the development of a normal fovea structure after birth can be observed using serial OCT images. With increasing age, a shallow foveal pit develops into a normal fovea via centrifugal migration of the inner retinal layer. The growth of photoreceptor cells also results in increased thickness of the retinal nerve fiber layer [20,22]. The interdigitation zone (IZ) is the last band on the OCT to be visible until 46 to 47 weeks of gestational age [20]. Perifoveal vessel density continuously increases with age and peaks at 10–15 years. [15]. In previous studies, the mean central macular thickness on OCT in children ranged from 258.6 to 274.968 µm [17,18,23,24] and the mean central macular volume in children ranged from 0.203 to 0.216 mm^3^ [18,23,24]. The outer segment of the retinal layer is 36% thinner than in adults [17].

Other studies also showed an increased peripapillary retinal nerve fiber (p-RNFL) layer thickness in 6–8-year-old children [25]. In another study by Yanni et al., in 83 healthy children aged 5–15 years, the peripapillary RNFL thickness was thicker than in White adults, particularly in the superior and inferior sectors [17]. This could be explained by the negative correlation of p-RNFL with age and development of the papillomacular bundle beyond 5 years old. A positive correlation between the RNFL and spherical equivalent was noted by Barrio-Barrio et al. in healthy Caucasian children aged 4–17 years old [26].

In healthy children, several parameters in OCTA images were investigated. In a study performed by Yang et al., in 570 Chinese adolescents aged 11 to 18 years old, the morphologies of the retinal microvasculature (deep capillary plexus perfusion density (DCP-PD), foveal avascular zone (FAZ) and perfusion vessel density (PVD)) were mainly influenced by sex rather than age [27]. A larger inferior hemiretinal superficial capillary plexus perfusion density (SCP-PD), DCP-PD, FAZ and PVD were noted in girls more than in boys [27]. In a study performed by Cheung et al., which included 1059 children aged 6–8 years, they observed that a thinner central macular thickness was associated with an enlarged FAZ area [13].

In another study by Zhang et al., which included 75 eyes from 75 Chinese patients aged 8–16 years, the authors showed that despite great inter-individual variation in FAZ areas, girls had a larger mean FAZ area than boys. [28]. A similar finding was noted in adults [29]. The exact mechanism of this finding is still unclear but gender differences in central retinal thicknesses and the spherical equivalent may play a role [29]. In a more recent study by Xiang et al., higher vessel densities in the SCP, DCP and radial capillary peripapillary (RPC) in boys were observed in preschool children aged 4–6 years old [30], which may be explained by increased serum insulin-like growth factor (IGF-1) in males that are 4–6 years old.

On the other hand, a study undertaken by İçel et al. showed that DCP vessel densities significantly decreased with increasing age but there were no significant gender differences in OCTA measurements [31]. In terms of ethnic differences, a larger FAZ was observed in African-American patients than in White patients [15].

Besides age and sex, refractive error and axial length are important factors that influence pediatric OCT findings. The pediatric myopic eye exhibited decreased average macular thickness and volume with a slightly increased foveal thickness in a study of Chinese children [32]. Cheung et al. also found that an enlarged FAZ area on OCTA was associated with decreased axial length, which the authors hypothesized to be due to increased circulatory efficiency [13]. Positive correlations between the axial length and the SCP, DCP and RPC were noted in 4–6-year-old preschool children during the emmetropization process [30]. A shorter axial length was also related to lower vessel densities on OCTA images [13], and similar findings were found for children with amblyopia [33,34]. The abovementioned findings could be explained by retinal or choroidal microvasculature alterations due to underuse in eyes with a shorter axial length [34]. Another study also found that a larger BMI was associated with decreased retinal vessel density, which was hypothesized to be influenced by a vascular structure change, including arteriole stiffness and resistance, and a reduction in microvascular density related to obesity [13,35]. OCTA metrics are not related to BMI in adolescence [27].

Suffice it to say, due to a wide variation in the pediatric age group, more studies are needed to establish a normative database, possibly based on age and ethnicity.

## 4. OCT and OCTA in Retinal Vascular Diseases

### 4.1. Retinopathy of Prematurity

**Figure 1:**
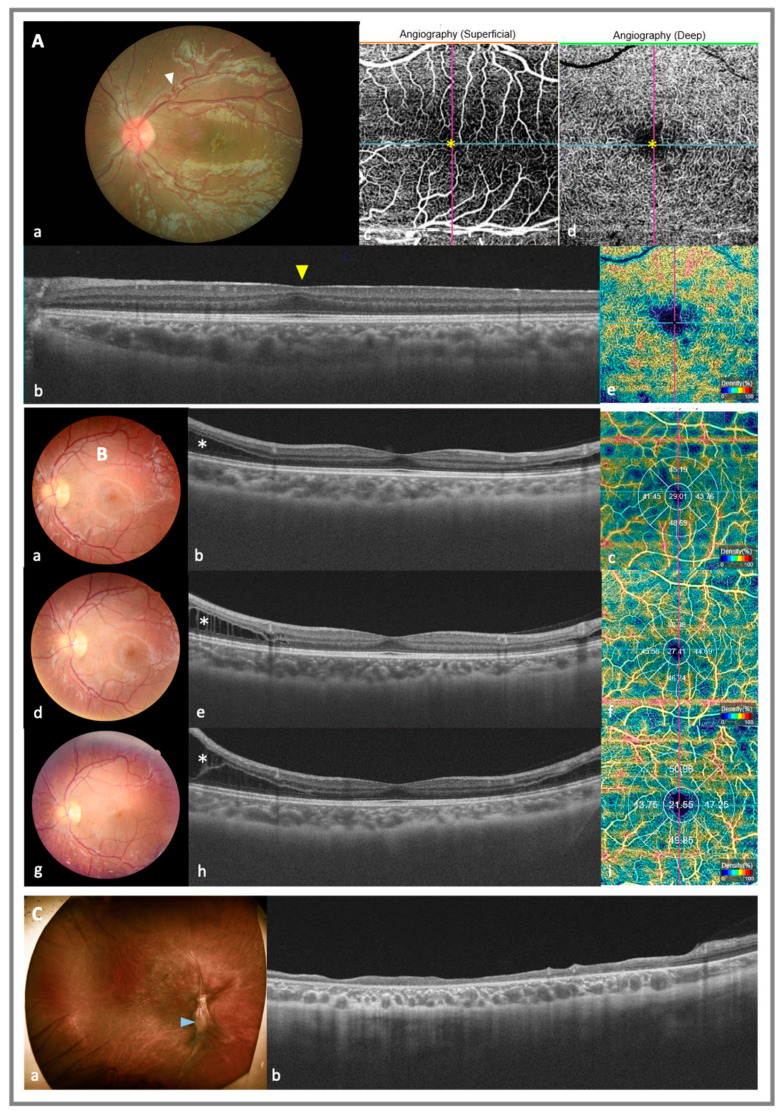
OCT and OCTA in retinopathy of prematurity (ROP). (**A**) A 6-year-old boy was previously diagnosed with stage 3 ROP with plus disease in both eyes and received an intravitreal bevacizumab injection at birth. He had a best corrected visual acuity (BCVA) of 1.0 in the right eye and 0.9 in the left eye. (**A**(**a**)) Color fundus photograph showing mildly tortuous retinal vessels (white arrow). (**A**(**b**)) Optical coherence tomography (OCT) image showing a loss of normal foveal depression (yellow arrow) consistent with foveal hypoplasia. (**A**(**c**–**e**)) Optical coherence tomography angiography (OCTA) image showing a small foveal avascular zone (FAZ) area (yellow asterisk) on the superficial capillary plexus (SCP) and deep capillary plexus (DCP). (**B**) Serial images in a boy post vitrectomy for stage 4A with plus disease in the left eye. He achieved a BCVA of 1.0 post-operatively. (**B**(**a**–**c**)) Slight splitting of the Henle’s fiber layer, or schisis (white asterisk), at the parafovea present at age 8. (**B**(**d**,**e**)) Cystic spaces increased at age 10 and (**B**(**g**,**h**)) at age 11, while the retina remained attached. (**B**(**c**,**f**,**i**)) OCTA density maps of the macula revealing a smaller FAZ than in **A**(**e**), and the foveal vessel density decreased over time. (**C**) A 5-year-old girl previously presented with stage 4A ROP with plus disease in the right eye and underwent lens-sparing vitrectomy at 2 months postnatal age. The post-operative BCVA was counting fingers in her right eye. (**C**(**a**)) Color fundus photograph showing attached retina with persistent fetal vasculature (blue arrow), (**C**(**b**)) while the OCT image reveals diffuse retinal thinning and atrophic change.

The current grading of retinopathy of prematurity (ROP) is dependent upon the stage and zone of disease, along with the presence or absence of “plus” disease [1]. This is primarily a clinical grading via indirect ophthalmoscopy. OCT has provided with us some new insights into the disease. A large number of studies investigated the use of OCT in ROP. First of all, OCT is able to identify subclinical structural changes that are overlooked by conventional indirect ophthalmoscopy (Figure 1). These findings include preretinal tissue (popcorn retinopathy), vitreous band, epiretinal membranes, cystoid macular changes, retinal layer schisis and foveal hypoplasia (Figure 1A) [36,37,38,39,40]. These can be demonstrated using a handheld device in very young premature infants or a tabletop OCT for older children [41,42]. Second, OCT may provide additional information for staging and prognostication of ROP upon diagnosis. For example, infants with clinical stage 4A may turn out to be stage 4B if OCT shows macular involvement, or vice versa; alternatively, infants may exhibit retinoschisis without retinal detachment in OCT images [43]. Third, abnormal vascular characteristics can be shown in OCT images, and the presence of vessel elevation, hyporeflective vessels and scalloped retinal layers may indicate plus disease [44]. Retinal morphology at the vascular–avascular junction and the fibrovascular ridge can be imaged with SD-OCT too [45,46]. One novel method described included extracting two-dimensional en face retinal vascular shadow view (RVSV) montage images from structural OCT scans, which can optimize visualization of the posterior pole vasculature by eliminating potentially distracting choroidal vasculature. RVSV-OCT images present with more distinct retinal vessels, better retinal vessel contrast and crisper margins than conventional ones, and thus, are gradable for plus, pre-plus or no plus disease [47,48]. Fourth, several studies showed that OCT was able to image a treatment response, such as regression of macular edema following laser or intravitreal anti-VEGF injection [49,50]. Nguyen et al. also described a rapid decrease in fibrovascular ridge thickness following intravitreal anti-VEGF injection in type 1 ROP [46]. For those children with advanced ROP receiving vitrectomy, OCT may show retinal re-detachment, retinoschisis or retinal atrophy during follow-up (Figure 1B,C). Fifth, microstructure–functional correlation was studied using OCT. Thinning of the retinal nerve fiber layer (RNFL) across the papillomacular bundle was associated with poor vision in preterm infants [51]. However, there have been no clinical trials that assessed the outcomes of ROP treatment driven by OCT diagnosis rather than using indirect ophthalmoscopy or wide-angle contact photography alone.

OCTA, on the other hand, can be applied for the investigation of microvascular changes, treatment response and structural–visual correlation in ROP as well. Most studies applied tabletop OCTA in older children with ROP, and they found significantly smaller FAZ areas (Figure 1A) in eyes with ROP than in controls [52,53,54]. This was negatively correlated with central retinal thickness [55], which was found to be higher in ROP [56]. In addition, high foveal VD with low parafoveal VD was reported [57,58]. Following treatment, the foveal VD decreased and was more significantly reduced in eyes treated with anti-VEGF injection than those in the laser group [59,60], which suggested that anti-VEGF treatment may result in better foveal structural development. Furthermore, although the visual correlation with several parameters on OCTA is yet to be shown [55], one study showed that a higher superficial vascular density was associated with suboptimal visual acuity [57]. As for the clinical availability of handheld OCTA in infants, it is still limited to some research prototypes [61,62,63].

There are certainly some limitations to OCT and OCTA. First, the image quality and quantitative analysis are usually affected by alignment errors and motion artifacts in children, which results in considerable data variability. Second, these modalities allow for a relatively limited field of view, which makes it difficult to image peripheral retina. Nevertheless, we believe that with the help of OCT and OCTA, the assessment of disease status will be improved, timely intervention can be taken, treatment response monitoring will be more precise and more outcome parameters can be quantified in the future.

### 4.2. Familial Exudative Vitreoretinopathy

Familial exudative vitreoretinopathy (FEVR) is an inherited disorder of retinal vascular development and is characterized by peripheral retina avascularity [64]. Pre-retinal neovascularization can develop and lead to macular and retinal traction or a retinal fold. The current staging system of FEVR is based on Pendergast and Trese’s study published in 1998 and is based on the extent of peripheral retinal vascularity, exudate and retinal detachment [65]. The progression of FEVR may not be stepwise based on the clinical stage, which makes FEVR a progressive disease that needs life-long follow-up [66,67]. This may eventually lead to severe complications, such as retinal detachment, a retinal fold and macula ectopia, which lead to blindness [64,68,69].

**Figure 2:**
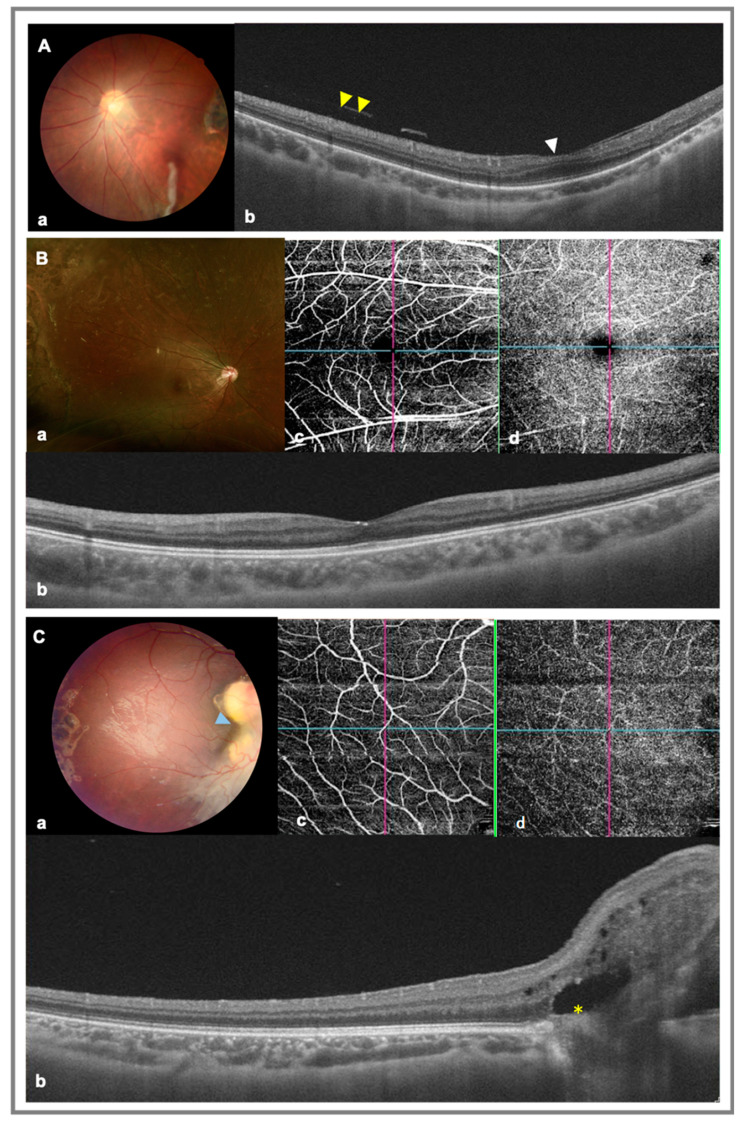
OCT and OCTA images of familial exudative vitreoretinopathy (FEVR). (**A**) A 26-year-old female with bilateral FEVR with a left eye best corrected visual acuity of 0.03. (**A**(**a**)) Color fundus photography of the left eye showing straightened vessels with peripheral avascular retina and two fibrovascular patches superior and inferior to the macula. (**A**(**b**)) The corresponding macula OCT shows a thickened posterior hyaloid (yellow arrow) and foveal hypoplasia (white arrow). (**B**) A 20-year-old woman with bilateral congenital cataract and right eye FEVR-related retinal detachment status after combined scleral buckling and pars plana vitrectomy when she was 11 years of age. The best corrected visual acuity was 0.7. (**B**(**a**)) Ultrawide field color fundus photograph of a right eye 9 years after surgery showing laser photocoagulation scars temporally. (**B**(**b**)) The corresponding macula OCT image showing foveal hypoplasia (white arrow). (**B**(**c**,**d**)) OCTA image showing a smaller FAZ on the superficial capillary plexus (SCP) and deep capillary plexus (DCP), and vessel straightening on the SCP slab. (**C**) An 8-year-old boy with a history of bilateral FEVR that received right eye laser photocoagulation temporally when he was 3 years of age. The best corrected visual acuity was 0.02. (**C**(**a**)) Color fundus photography of the right eye showing a fibrovascular stalk at the nasal retina and optic disc causing optic nerve head dragging (blue arrow). (**C**(**b**)) The corresponding macula OCT image showing vitreopapillary traction, a subpapillary cystic space (yellow asterisk) and foveal hypoplasia. (**C**(**c**,**d**)) OCTA image showing the absence of the FAZ and decreased vessel density in the SCP and DCP.

An optical coherence tomography finding of FEVR can provide information about the posterior vitreous and structural changes of the retina (Figure 2). OCT was able to visualize posterior hyaloidal disorganization, which ranged from a thin epiretinal membrane to thicker vitreomacular or vitreopapillary traction [64]. A disorganized posterior hyaloid is more common in higher stages of FEVR [70]. Persistent inner foveal fetal architecture or foveal hypoplasia, macular edema, atrophy of the outer retina (mainly the ellipsoid zone (EZ)) and retinal detachment were identified using OCT on FEVR patients [42,64]. Greater foveal thickness and central macular thickness; microstructural change, including posterior hyaloidal disorganization; vitreomacular traction; macular edema; and EZ disruption are correlated with poorer visual acuity in stage 1 and stage 2 FEVR [64]. A thicker central retinal thickness (CRT) can be attributed to a residual retinal inner layer and the absence of a foveal pit [71,72]. A recent publication by Zhang et al. described using ultra-wide-field laser ophthalmoscopy and optical coherence tomography to identify a novel sign of temporal mid-peripheral vitreoretinal interface abnormality (TEMPVIA) in early-stage FEVR, which was characterized by retinal thickening at the posterior margin of avascularized retina, with mild or absent retinoschisis and leakage on the FA [73]. Outer retina atrophy as seen using OCT was pointed out to be an independent risk factor for poor visual acuity [64]. Lee et al. applied handheld OCT to children with FEVR (mean age 32 months) and found temporal and anterior retinal displacement, prominent vitreopapillary adhesion and RNFL thickening at the optic nerve head margin [69]. OCT-vitreopapillary dragging (OCT-VPD) was observed in 16 eyes with nearly normal fundus appearances but optic nerve head elevation was detected using OCT [69]. This finding could explain the clinical finding of optic nerve head dragging and fibrovascular band in FEVR patients via tangential and circumferential preretinal neovascularization toward peripheral retina, as well as anteroposterior vitreous traction [69].

Conventional FA imaging of FEVR was able to show peripheral avascularized areas, abnormal venous–venous anastamosis, vascular branching and leakage in the peripheral retina [73]. In terms of OCTA of FEVR, central macular microvascular change was noted in several studies, which suggested that there may be more to FEVR than peripheral retinal avascularization [67,71,72]. Hsu et al. reported a series of 11 eyes of 6 patients with FEVR imaged with OCTA, which showed dilation, disorganization, straightening, heterogeneous vessel density and curls formation in SCP [67]. Decreased vessel density, disorganization of vessels, curls/loops and a specific finding of end-bulbs in 7 out of 11 eyes were noted in the DCP of an FEVR patient [10,67]. These findings were minimal on traditional fluorescein angiography. The end-bulbs in the DCP on OCTA images may correlate with structural vessel termination, which indicates defective retinal angiogenesis in the deep retinal layer in FEVR pathogenesis [67]. Another series by Koulilis et al. analyzed 187 eyes in 117 patients and showed significantly lower VD and an increased vessel diameter index (VDI) in patients with stage I and stage II FEVR in comparison with an age-matched control group in the nonsegmented retina, superficial retina and deep retina [74].

Regarding the FAZ, some earlier studies showed smaller FAZs in FEVR patients in comparison to controls; Hasegawa et al. found that in stage I or II FEVR, both the surface and deep FAZ areas (0.265 ± 0.08 and 0.364 ± 0.1 mm^2^, respectively) were smaller in the FEVR group than in the control group [71]. Chen et al. also noted a smaller FAZ (0.27 ± 0.17 mm^2^) in 31.70% of FEVR eyes (13 out of 41) but not in controls [72]. However, in the largest series by Koulilis et al., the FAZ was larger only in the deep retina layer but not in the superficial retina layer in stage I and II FEVR, and the size of the FAZ was not correlated with visual acuity [74].

A more recent study by Mao et al. also found lower VD of the DCP in the digenic inheritance of FEVR patients. Patients with LRP5 variants had lower foveal VD in SCP and DCP and a higher acirculatory index than TSPAN12 variants [75]. This finding shows the application of OCTA in different phenotypes and genotypes in the FEVR disease spectrum. Using the early detection of structural changes and identifying subtypes of FEVR, ophthalmologists may be able to provide more individualized and precise treatments, adopt early surgical or medical interventions in FEVR patients, and better evaluate disease prognoses.

### 4.3. Coats Disease

Coats disease is an idiopathic congenital retinal vasculopathy that mainly comprises telangiectasia that results in exudation, exudative retinal detachment or macular fibrosis. Classification and diagnosis are based on clinical examination and fluorescein angiography. Coats disease typically develops in young males in the first or second decade of life [76]. The classification of Coats disease is based on clinical findings developed by Shields et al. in 2001 [77].

**Figure 3:**
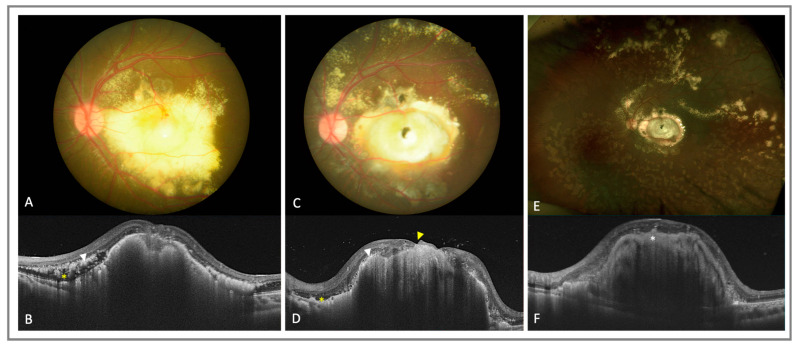
OCT images showing Coats disease. A 7-year-old boy presented with Coats disease in the left eye with a BCVA of 0.06. (**A**) Color fundus photography showing a massive macular exudation and telangiectatic vessels. (**B**) OCT image showing subretinal fluid (yellow asterisk) and intra-retinal hyper-reflective materials (white arrow) corresponding to exudates. (**C**) One year after multiple sessions of intravitreal bevacizumab and laser photocoagulation treatment, the exudation partially resolved and became more consolidated with a macular scar formation. (**D**) OCT image showing the submacular exudation breaks through the internal limiting membrane, with a reduction in subretinal fluid and hyper-reflective materials (white arrow). (**E**) Two years after treatment, the macular scar became more well-defined and the mid-peripheral laser scars were visible on ultra-widefield imaging. (**F**) The corresponding OCT image showing subretinal fibrosis and organization (white asterisk) with the resolution of subretinal fluid, intra-retinal cysts and hyper-reflective dots, which indicates reduced activity.

With the development of OCT and OCTA, subtle changes related to early Coats disease can be detected. For example, intraretinal edema or intraretinal exudation was noted using SD-OCT of stage 2b disease [78]. In a retrospective cohort study of 29 eyes in 28 children published by Ong et al. in 2019, OCT imaging of Coats disease found that the highest density of exudates was in the upper half of the outer nuclear layer (ONL), followed by the subretinal space. OCT was able to image macular subretinal fluid (SRF) and possible small pockets of SRF that may not be visible on a color fundus photo or clinical examination. The authors also found small hyper-reflective dots in areas without exudates; these hyper-reflective dots were speculated to represent macrophages within exudates [79]. Outer retinal tubulations that represented degenerating photoreceptors were also noted [79]. In another multicenter, retrospective cohort study by Gupta et al. that included 27 eyes from 27 cases, OCT helped in the identification of microstructural retinal changes, such as intraretinal edema and SRF, vitreoretinal interface abnormality, EZ and external limiting membrane (ELM) disruption [78]. Microstructural findings of OCT image features associated with poor visual prognosis include subfoveal fibrosis, atrophy and subfoveal nodules in early-stage Coats disease [2,80] (Figure 3). Increased foveal total retinal thickness, central subfield thickness and macular volume were associated with poor visual outcomes in Coats disease [81]. Abnormalities listed by Gupta et al. showed that intraretinal exudate, subretinal fluid, subretinal exudate, ellipsoid zone disruption and external limiting membrane disruption using initial OCT were associated with both poorer initial and final visual acuities [78]. The case with ellipsoid zone disruption had the poorest initial and final visual acuities (logMAR 1.26 and 1.12, respectively, in comparison to VA logMAR 0.35 and 0.19 in cases without EZ disruption, respectively) [78]. A longitudinal follow-up showed that cases that presented with a subfoveal nodule at the initial SD-OCT had a significantly higher risk of having a macular scar at the follow-up [78]. A hyper-reflective lesion extending from the Bruch membrane/RPE layer to the inner retina may cause macular fibrosis [82]. Furthermore, ILM wrinkling resulting from fibrosis-induced retinal traction was also observed using OCT [82]. These findings indicated that early identification of microstructural change using OCT may help a clinician to decide the optimal treatment and better refine patients’ visual prognosis.

OCTA of Coats disease was also able to identify telangiectasia and capillary tortuosity in the DCP and SCP. Traditional FA images showed telangiectatic vessels with a leakage or cystoid macular leakage [79]. On the OCTA images, obliteration or absence of a FAZ by irregular, coarse vessels in retinal plexuses, along with chorioretinal anastomosis, may be noted [82]. Schwartz et al. found a decreased VD in the SCP and DCP in the affected eye when compared with the fellow eye in 13 cases with unilateral Coats disease [83]. Quantification of macula vascular density and the FAZ area was also proposed by Zhang et al. [84], with a lower vascular density also noted in patients with refractory macular edema [84]. A higher percentage of telangiectasia (76.9%) was also found in the DCP in comparison to SCP (34.6%) [78]. This finding led to the DCP being postulated to be the first layer involved in Coats disease. This may be due to the vascular supply of the SCP and DCP, with the DCP being distal vessels with weaker walls and more likely to be disrupted in Coats disease [84]. In another series by Cennamo et al., the authors noted capillary hypoperfusion with an increased FAZ area in the SCP, along with irregularly dilated small perifoveal vessels and a loss of collateral branches and increased vascular rarefaction, were noted in the DCP [85]. In eyes with macular fibrosis, a disrupted vascular network and coarse macula vessels may be observed using OCTA [82]. However, it is important to note that subretinal exudate may lead to obscuration of choriocapillaris (CC), and manual segmentation of OCTA images in a patient with macula exudates may be required to accurately visualize blood vessels [84,85]. In addition, OCTA of Coats disease may also reveal an irregular FAZ with crossing vessels on both the SCP and DCP [10]. A study performed by Cennamo et al. regarding treatment response monitoring using OCT and OCTA after serial intravitreal anti-VEGF in Coats disease in 15 eyes in 15 patients with a mean age of 20.4 ± 2 years also showed subretinal exudates persisted in the macular region and generalized vascular rarefaction was still noted in the SCP, DCP and CC [85]. Overall, by identifying subtle changes using OCT and OCTA, which guided the treatment strategy and monitoring of the treatment effect, the above study showed potential for the application of these novel imaging modalities in Coats disease [78].

### 4.4. Incontinentia Pigmenti

Incontinentia pigmenti (IP) is an X-linked dominant inherited disease caused by a mutation in the IKB-KG/NEMO gene that predominantly affects the peripheral retina [86]. It often manifests as vascular occlusion, peripheral retinal nonperfusion, neovascularization and subsequent retinal detachment. OCT of the macula can detect structural subnormalities, such as focal inner retinal thinning and an outer plexiform layer irregularity [87,88]. Handheld SD-OCT imaging revealed additional information in very young neonates that ranged from a normal retinal structure to inner and outer retinal thinning [89]. Pathologic changes found using OCTA include decreased VD, abnormal vascular loops, and regions of flow loss in the SCP and DCP, mostly temporal to the fovea [88]. Several case reports also revealed a non-perfusion area with a small FAZ in the SCP and DCP [90,91]. Hence, in a child diagnosed with IP with normal fundus examination, SD-OCT and OCTA can still detect a subtle abnormal microstructure and changes in vascular flow, respectively [92]. These findings may explain the visual impairment in some IP patients without tractional retinal detachment. The potential association between macular and peripheral retinal pathology can also be studied in the future. Currently, technology limits the field of view of OCTA (especially handheld versions). With the expected development of widefield OCTA for children, it is possible that we will be able to perform OCTA-guided (instead of FA-guided) laser photocoagulation of the peripheral avascular retina.

### 4.5. Type-I-Diabetes-Mellitus-Related Retinopathy

In pediatric patients with type 1 diabetes (T1D), diabetic retinopathy (DR) usually presents 8 to 10 years after diagnosis [93]. The prevalence of DR mostly ranges from 2% to 10% in the pediatric population and increases with age [94]. Most studies shed light on the early detection of subclinical retinal changes in T1D. OCT was applied in children with T1D and showed an increased central choroidal thickness and thinning of the inner retina even before the development of DR [95,96,97]. The thickening of the choroid may reflect a regulatory attempt to increase perfusion in the presence of ischemia [98]. Thinning of the inner retina could imply a higher perfusion demand that makes it more vulnerable to diabetic-induced metabolic stress [99]. Not surprisingly, decreased vascular density in the SCP and DCP with enlargement of the FAZ was also noted in the OCTA images of patients with T1D compared with healthy subjects [100,101,102]. All the above findings are manifestations of the beginning of diabetic choroidopathy and retinopathy. In T1D patients with DR, a greater HbA1c level was also found to be associated with increased central retinal thickness [103]. In addition, the duration of diabetes and the severity of DR were positively correlated with an increased FAZ area and negatively correlated with the VD at the SCP [104,105,106]. Perhaps OCT and OCTA can be used as screening tools in children with T1D for early detection of DR and subclinical disease progression. The management of DR in children is no different than in adults and will depend on the disease severity.

## 5. OCT and OCTA in Inherited Retinal Degenerations

### 5.1. X-Linked Juvenile Retinoschisis

X-linked juvenile retinoschisis (XLJR) is an inherited bilateral vitreoretinal degeneration caused by mutations in the RS1 gene, which encodes the retinoschisin protein [107]. It is characterized by the splitting of the inner retinal layer, including the RNFL and ganglion cell layer. Widespread cystic spaces and disorganization of the retinal layers around the fovea are the most classic findings using OCT. Based on an OCT scan, XLJR is further classified into four phenotypes [108]. In addition, OCT can reveal areas of schisis that may not be visible on fundus examination [108]. Some studies even showed retinal splitting in layers other than the nerve fiber layer [109,110,111]. Moreover, a loss of photoreceptor integrity, decreased density in the cone outer segment tips, or defects in the ELM and EZ were identified using FD-OCT and swept source (SS)-OCT, and these outer retinal abnormalities were shown to be associated with poor vision [109,112,113,114]. Nowadays, handheld OCT has enabled us to image infants and young children [115]. The pattern of XLRS in adults can be present as early as in young childhood, suggesting early screening and diagnosis are warranted in high-risk patients [116].

OCTA for XLJR may reveal microvascular irregularities or a petaloid pattern at the superficial and deep vascular plexuses due to the presence of schisis at the INL [109]. An irregular and enlarged FAZ accompanied by reduced macular deep vessel density was observed compared with control eyes [117,118,119]. Kwon et al. evaluated these structural changes and functional outcomes in their study and showed that foveal flow loss in deep capillary plexus, foveal avascular zone enlargement, thinner inner retina layer and disorganized photoreceptor layer was positively correlated with a decline in BCVA [119,120]. Mastropasqua et al. found increased deep capillary plexus density after treatment with acetazolamide and dorzolamide in patients with XLJR, suggesting OCTA can be used as a tool for monitoring treatment response [118]. Therefore, OCT and OCTA may prove to be important in clinical practice not only because of their role in diagnosis but also the potential for monitoring the treatment response of XLJR.

### 5.2. Stargardt Disease

Stargardt disease (STGD) is the most common childhood recessively inherited macular dystrophy and is caused by mutations in the ABCA4 gene [121]. It is characterized by bilateral loss of central vision presenting from early childhood due to abnormal lipofuscin accumulation at the photoreceptor level [122]. SD-OCT can reveal abnormal subretinal flecks presenting as hyper-reflective deposits that begin at the central macula, which gradually disrupts the IZ, EZ, ELM and ONL [123,124,125]. ELM thickening is thought to be an early sign that presents in pediatric patients and may precede changes on fundus examination [125,126,127]. SD-OCT and en face OCT also provide quantitative measurements of the mean retinal thickness and volume and can be utilized in monitoring disease progression [128,129,130]. Unsurprisingly, the mean central foveal thickness and total macular volume were found to decrease over time [131,132], and quantification of the EZ defect and RPE atrophy on serial SD-OCT scans were found to be predictors of progression [133,134].

On the other hand, OCTA was applied to study retinal and choroidal alterations in STGD. It was suggested that vascular changes may play an important pathogenic role in STGD, as choriocapillaris atrophy is commonly related to RPE atrophy [135,136]. OCTA in eyes with STGD had reduced VD of the SCP, DCP and CC compared with healthy subjects [136,137] and the FAZ was larger [138]. The extent of choriocapillaris VD loss was reported to be correlated with the magnitude of RPE and photoreceptor degeneration, decreasing parafoveal macular thickness and declining BCVA [130,139]. A vessel tortuosity cutoff of 5 in OCTA images was proposed as a parameter to distinguish the two STGD phenotypes, namely, STGD1 and STGD2, with different visual outcomes [140]. Another study found different vascular characteristics among patients with inherited retinal diseases, including STGD, retinitis pigmentosa and cone–rod dystrophy [141]. One multicenter study used an OCTA-measured choroidal vascularity index to differentiate central macular atrophy secondary to STGD or age-related macular degeneration [141]. The authors hypothesized that large choroidal vessels were impaired to a greater extent in AMD than in STGD.

When imaging patients with Stargardt disease, one limitation to be aware of is that the presence of eccentric fixation may reduce the test quality, repeatability and reproducibility [142]. There is currently no approved treatment for Stargardt disease, and we await the results of gene therapy trials. Although OCT and OCTA may not be able to change the treatment of STGD currently, they provide more information on the structural and vascular pathologies that might aid patient selection for gene therapy in the future.

## 6. OCT and OCTA in Neoplasm

Among all pediatric ocular tumors, retinoblastoma (Rb) is the most common ocular malignancy, which can lead to permanent visual impairment and mortality. The diagnosis is usually made clinically, with leukocoria being an important sign. However, Rb must be differentiated from other conditions, such as Coats disease, persistent hyperplastic primary vitreous or astrocytic hamartoma. OCT can obtain cross-sectional images and aid in the diagnosis of Rb. For example, unlike retinal astrocytic harmartoma, which arises from the RNFL, the dome-shaped retinal thickening appearance of retinoblastoma mainly involves the middle or outer retinal layers [143,144]. In addition, tumor calcification can present as high internal reflectivity in OCT images [145]. Preretinal seeds in the posterior pole and subretinal fluid or seeds are also clearly imaged with OCT [146]. These findings were successfully shown on a handheld system [144,145,146,146,147] and used for the detection of clinically invisible smaller tumors [148,149].

Furthermore, OCT or OCTA scans can nowadays impact Rb treatment decisions and direct follow-up plans [150]. Nadiarnykh et al. pointed out some features of active and inactive Rbs using SD-OCT [145]. The isodense appearance mass became more variably dense or flattened in response to treatment [146]. The resolution of subretinal fluid and maculopathy following chemotherapy can be precisely detected [151]. With the advances in SS-OCT technology that enabled a deeper penetration into the tumor, Damodaran et al. described three different regression patterns of Rb [152]. This allows for better follow-up of Rb in order to detect early recurrences. Small lesions away from the fovea had typically regressed, leaving a depressed and completely atrophic retinal tissue. Larger central lesions had poor visibility of the outer retina in OCT images and regressed with either a smooth homogeneous appearance or an irregular heterogeneous appearance.

OCTA was recently implemented to understand how the retinal microvasculature changes following treatments. It was found that the intrinsic tumor vascularity decreased and the draining and feeder vessels became less dilated after transpupillary thermotherapy [153]. The microvascular features corresponded with the pattern of regression and persistently dilated feeder vessels may represent active disease [154,155]. Early or atypical recurrence of Rb can be detected with the help of these images [156,157]. As for the long-term impact on eyes after intravenous chemotherapy, lower deep capillary densities were observed using OCTA, while the retinal thickness, FAZ, superficial VD and choroidal thickness remained unaffected [158,159].

Specific limitations of OCT and OCTA in imaging Rb include the need for a transparent media, and some far peripheral lesions are not accessible. Ultrawide-field OCT was put forward as a possible solution to circumvent these problems but is currently at the prototype stage [160]. Despite the limitation, we believe these images will benefit pediatric patients with Rb at initial evaluation and subsequent follow-up visits.

## 7. OCT and OCTA in Inflammatory/Uveitic Diseases

OCT was widely applied in pediatric uveitis. For example, even though JIA-related uveitis is usually confined to the anterior segment, it was found that macular edema and foveal detachment may be seen using OCT. Moreover, an anterior chamber reaction can be imaged with anterior segment-OCT (AS-OCT) to show cells in the anterior chamber as hyper-reflective loci [161]. OCT can also detect cystoid macular edema in the pars planitis and intraretinal fluid, ERM and epiretinal membrane, intraretinal fluid and optic nerve edema in tubulointerstitial nephritis and uveitis (TINU) syndrome, as well as sub- and intraretinal fluid and choroidal undulation in Vokt–Koyanagi–Harada (VKH) disease [161]. Increased subfoveal choroidal thickness was also noted in Behçet disease [162].

Quantitative OCTA showed significantly decreased SCP and DCP vessel density in eyes with pediatric uveitis, regardless of disease activity [163]. Similar findings were also shown in JIA-related uveitis in 38 eyes from 20 Egyptian children (with a mean age of 10.7 ± 2.6 years); in moderate-to-severe-activity JIA-related uveitis patients, significantly lower VD in the SCP and DCP, increased FAZ and decreased central macular thickness were noted [164]. In active pediatric anterior uveitis, the subfoveal choroidal thickness (SFCT) was increased in comparison to inactive and control patients but the FAZ and central macular thickness (CMT) were not significantly different [163]. The above findings suggest subclinical inflammation of the posterior segment in pediatric anterior uveitis. In a recent study by Tuğan et al. of 22 eyes from 22 pediatric patients (with a mean age of 13.75 ± 2.25 years), OCTA could also detect microvascular changes in pediatric Behçet disease without ocular involvement, a significantly decreased DCP but not SCP vessel density and decreased outer retinal flow were observed; the FAZ had no significant difference from the healthy controls [165]. These applications of OCTA showed that this noninvasive examination may serve as a tool for early microvascular monitoring in possible systemic disease-related pediatric uveitis and may also evaluate disease activity. However, it is noteworthy that OCTA can only visualize the structure of the vascular network rather than showing the permeability or leakage of vessels as per FA or ICGA [5], which may limit its use in evaluating posterior uveitis. Based on OCT and OCTA findings, pediatric uveitis activity may be monitored and detected early via the prompt initiation of topical or systemic treatment, and possible vision-threatening complications, including band keratopathy, cataract, uveitic glaucoma or macula scarring, may be prevented.

## 8. OCT and OCTA of Miscellaneous Conditions

### 8.1. Nonaccidental Traumatic Brain Injury (NAI) or Shaken Baby Syndrome (SBS)

Pediatric abusive head trauma, also known as nonaccidental traumatic brain injury (NAI) or shaken baby syndrome, can present with ocular findings, the most pathognomonic being multiple layered retinal hemorrhaging [166]. OCT was shown to identify vitreoretinal abnormalities, including posterior vitreous detachment, multilayered retinoschisis, foveal structure disruption, disinsertion of ILM, macular hole and perimacular folding [20,166,167]. These findings help the clinician identify the signs of retinal structural change and may guide subsequent surgical intervention.

In a recent case report by Echegaray et al., OCTA findings in a boy with NAI showed parafoveal attenuation of the SCP, intermediate capillary plexus (ICP) and DCP but an intact peripapillary vascular plexus [168]. This showed that OCTA can be used to detect macular ischemia in NAI.

### 8.2. Torpedo Maculopathy

Torpedo maculopathy is a rare benign congenital defect of the retinal pigment epithelium. It is usually unilateral and presents as a hypopigmented oval nevus-like lesion in the temporal fovea [169,170].

OCT can be applied to classify torpedo maculopathy lesion types based on a pattern of attenuation of outer retinal structures, presence of outer retinal cavitation and neurosensory elevation [170]. The clinical significance of this is that excavation of the fundus results in a corresponding scotoma. Other OCT findings of torpedo maculopathy include hyper-reflective transmission into the choroid, atrophy of the outer retina, disruption of the ellipsoid zone and of the retinal pigment epithelium layer, and possibly neurosensory detachment [169,171]. Despite its benign nature, the development of the choroidal neovascularization (CNV) membrane was noted in torpedo maculopathy [170].

In a series published by Giannakaki-Zimmermann et al., lesions of torpedo maculopathy can be quantified using OCTA. In all four cases of the series, OCTA showed normal superficial and deep vascular capillary beds in the lesion area. However, the extension of the lesion into choriocapillaris and attenuation of the choriocapillary vascular network were noted in some patients [171].

## 9. Challenges and Future Directions

With a growing body of evidence supporting the use of OCT and OCTA in pediatric retinal diseases, there are opportunities to integrate these imaging modalities into routine clinical care. Table 1 summarizes the pertinent OCTA and OCTA findings from the literature. However, some issues remain unresolved.

First, Lim et al. found using handheld OCT that the average ganglion cell complex and retinal nerve fiber layer thickness was stable from 6 months to 5 years of age and established a normative database [172]. However, this study was performed with a predominantly White population, with a sample size of 67. Hsu et al. also found using OCTA that age, race and axial length are factors that influence quantitative parameters when imaging the pediatric perifoveal vasculature [15]. Image optimization is needed in pediatric eyes due to increasing axial length with age, evolving refractive status, steeper cornea and greater astigmatism in the first 6 months of life [9]. Moving forward, more work is needed to validate different portable OCT and OCTA over populations of various ethnicities. There is also a need for a quantitative age-adjusted pediatric normative database for retinal thicknesses on OCT [2].

Second, in terms of longitudinal follow-up, current handheld systems are also unable to perform image registration, which may be important in following response to treatment, especially in poorly co-operative patients. OCTA requires re-scanning the same retinal position multiple times, and thus, requires stabilization during examination [5]. Until then, imaging using handheld OCT and/or OCTA will be highly operator dependent. Even pediatric retina experts report having a learning curve associated with a handheld OCT device [9]. Eye-tracking capabilities and a faster acquisition time will also be helpful in less cooperative children [11]. Moreover, in conditions such as ocular albinism or nystagmus due to poor vision, saccadic eye movements may limit the ability to obtain good-quality OCT images.

Third, current handheld OCT systems may not have inbuilt automated OCT segmentation and thickness map software, but is available on the investigational Heidelberg Spectralis Flex module (Heidelberg Engineering, Heidelberg, Germany) [2]. However, the Heidelberg Spectralis Flex module may be bulky and cumbersome to be used in the NICU setting. Future research should focus on improving software capability (which can be aided by more work on establishing a normative database) and reducing the overall bulk of the imaging system. AI may help in automated image segmentation and quantitative analysis of OCT and OCTA images [173,174]. Deep-learning-based models were found to also help in classifying disease, monitoring prognosis, predict functional changes and providing image quality control [175]. In the future, there is potential for the implementation of AI in OCT/OCTA images to aid in disease diagnosis and monitoring. For example, the combination of OCT and OCTA parameters using AI was found to improve the classification of diabetic eyes [176].

The implementation of artificial intelligence (AI) in pediatric retinal diseases was mostly based on color fundus photography, especially in the field of ROP. Imaging and informatics in an ROP deep learning (i-ROP DL) AI system was established for screening, diagnosis, staging and assessing the vascular severity score in ROP patients [177]. More work is needed in evaluating AI in OCT and OCTA for pediatric retinal disease. Challenges to the application of a DL system in pediatric retinal disease include the lack of standardized images, noise in images, artifacts during image acquisition and the smaller sample size in patients with OCT/OCTA in comparison with color fundus photography [175]. Although AI may be an exciting new frontier, it has inherent issues in implementation, such as sustainability, cost-effectiveness and scalability [178,179].

**Figure 4:**
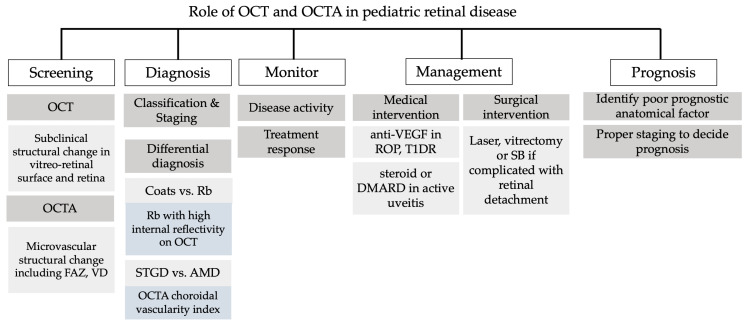
Proposed flowchart for OCT and OCTA in pediatric retinal disease. Abbreviations—OCT: optical coherence tomography, OCTA: optical coherence tomography angiography, Rb: retinoblastoma, STGD: Stargardt disease, AMD: age-related macular degeneration, VEGF: vascular endothelial growth factor, ROP: retinopathy of prematurity, T1DR: type 1 diabetic retinopathy, DMARD: disease-modifying anti-rheumatic drugs, SB: scleral buckle.

Fourth, it is also uncertain how the use of OCT and OCTA in pediatric retinal diseases may change our treatment paradigm. Currently, as OCT and OCTA are not in widespread clinical use in pediatric retina practices (due to their scarcity and lack of commercial devices), there is an issue with the interpretation of images. Does knowing that the ridge thickness is decreasing using OCT after anti-VEGF for ROP value add to traditional clinical evaluation of resolving “plus” disease and regression of stage 3 as seen via indirect ophthalmoscopy? Will knowing that a stage 4A ROP has retinoschisis change our surgical management (or prognosis)? Even then, the incidence of advanced ROP is low and does that warrant an expensive technology? Prior to the application of new technology into a clinical workflow system, a thorough health technology assessment also needs to be performed. From reviewing current evidence (which can be achieved with review articles such as this) to economic and budget evaluation to assessment of the social, ethical and legal considerations [180]. These are critical steps that one should consider in this era of burgeoning healthcare expenditure. We propose a flowchart for clinicians to potentially adopt OCT and OCTA in the evaluation of pediatric retinal disease (Figure 4).

Fifth, there is a shortage in the health workforce for pediatric ophthalmology and pediatric retinal care [1,9], which may limit training in using these sophisticated technologies. There may also be issues with parental consent if there are concerns about novel imaging devices causing possible discomfort to infants while not adding much value to clinical care, albeit OCT and OCTA are non-contact imaging techniques. Consent-taking and explanation may be a lengthier process compared with an adult patient [9].

Sixth, in terms of technological development, current OCTA devices were reported to have a limited dynamic range for flow velocity [5], i.e., the output saturates at a low flow rate. Improving the quantification of low flow rates will improve the identification of lesions, such as macular neovascularization, and be better able to monitor the response to treatment. Furthermore, many of the pathologies related to pediatric vascular retinal disease, such as ROP, FEVR and Coats disease, are in the peripheral retina. Developing widefield OCT and OCTA capabilities is imperative to identifying and monitoring disease activity.

Last, more research is needed in developing new imaging techniques and in exploring novel applications of OCT and OCTA in the pediatric population. For example, regarding the biallelic RPE65 mutation, which is the first inherited retinal degeneration with an FDA-approved gene therapy treatment, OCT was shown to identify patients with relative preservation of foveal photoreceptors and hence these patients have a higher likelihood of achieving rescue of central vision with gene therapy [2]. In the future, integrating OCT and OCTA data with other clinical and genetic information may help in candidate selection for future gene therapy trials, which can improve disease diagnosis and management.

## 10. Conclusions

In conclusion, currently, both OCT and OCTA are not considered to be mainstream imaging modalities in the evaluation of the pediatric retina. However, ongoing research revealed numerous potential uses, although their clinical application remains to be seen. This review showed that OCT and OCTA can reveal valuable information about various pediatric retinal diseases, which can also stimulate ideas for future research. We believe that with both software and hardware technological enhancements of OCT and OCTA, improved normative databases and more research into other pediatric retina conditions, OCT and OCTA will be a valuable addition to the ophthalmologist’s armamentarium in evaluating the pediatric retina.

## Figures and Tables

**Table 1:** Summary of the OCT and OCTA findings and metrics in various pediatric retinal diseases.

Diseases	OCT		OCTA	
	Thickness	Structures	FAZ	VD
Vascular Diseases				
ROP	Increased central foveal thickness, representing foveal hypoplasia Decreased choroidal thickness	Preretinal tissue Vitreous band Epiretinal membranes Cystoid macular changes Retinal layer schisis Elevated, hyporeflective or scalloped retinal layers representing plus disease	Reduced	Increased foveal VD Decreased foveal VD after treatment
FEVR	Increased	Posterior hyaloidal disorganization Foveal hypoplasia Macular edema Atrophy of the outer retina TEMPVIA on early stage FEVR Vitreopapillary dragging/adhesion	Reduced in most studies Koulilis et al. found FAZ larger only in the deep retina layer in stage I and II FEVR	Decreased foveal VD in the SCP and DCP Disorganization of vessels End-bulbs in DCP
Coats disease	Increased	Intra- and subretinal exudates Subretinal fluid Outer retinal tubulations Subfoveal fibrosis, atrophy and nodule Outer retina disruption ILM wrinkling	Reduced, even absence or obliteration may accompany crossing vessels in the FAZ	Decreased VD in the SCP and DCP Irregularly dilated small perifoveal vessels Loss of collateral branches in the DCP Increased vascular rarefaction in the DCP
IP	Normal or focal inner or outer retinal thinning	Outer plexiform layer irregularity Tractional retinal detachment in severe cases	Reduced	Decreased VD Abnormal vascular loops Temporal flow loss in the SCP and DCP
Type 1 DR	Increased central choroidal thickness at the early stage Increased central retinal thickness corresponds with higher HbA1c	DME, epiretinal membranes, vitreoretinal traction, hyper-reflective foci	Enlarged	Decreased VD in the SCP and DCP
Inherited Diseases				
XLJR	Increased	Splitting of the inner retinal layer with cystic spaces Loss of photoreceptor integrity Decreased density of cone outer segment tips	Irregular and enlarged	Microvascular irregularities with petaloid pattern in the SCP and DCP Flow loss in the DCP Increased DCP after treatment
STGD	Decreased central foveal, parafoveal thickness and total macular volume over time	Hyper-reflective deposits represent subretinal flecks IZ, EZ, ELM and ONL disruption	Enlarged	Reduced VD of the SCP, DCP and CC
Neoplasm				
Rb	Increased, dome-shaped	High internal reflectivity represents calcification Preretinal seeds and subretinal fluid at diagnosis Depressed and atrophic retinal tissue after treatment	Unaffected after treatment	Reduced deep capillary densities after treatment Intact superficial VD after treatment
Inflammatory Disease				
JIA-related uveitis	Decreased in moderate/severe disease	AS-OCT: hyper-reflective foci indicate cells in the anterior chamber Macular edemaFoveal detachment	Enlarged in moderate/severe disease	Decreased VD in the SCP and DCP
Miscellaneous				
NAI	Increased due to hemorrhage	Posterior vitreous detachment Multilayered retinoschisis Foveal structure disruption Disinsertion of ILM Macular hole Perimacular folding	N/A	Parafoveal attenuation of the SCP, ICP and DCP
Torpedo maculopathy	Usually normal, may decrease due to atrophic change	Type 1: outer retina attenuation Type 2: outer retina attenuation with outer retinal cavitation and neurosensory elevation or detachment	N/A	Normal SCP and DCP in the lesion area. Extension of the lesion into CC Attenuation of the CC vascular network

Abbreviations—OCT: optical coherence tomography, OCTA: optical coherence tomography angiography, FAZ: foveal avascular zone, VD: vessel density, ROP: retinopathy of prematurity, SCP: superficial capillary plexus, DCP: deep capillary plexus, FEVR: familial exudative vitreoretinopathy, IP: incontinentia pigmenti, DR: diabetic retinopathy, DME: diabetic macular edema, XLJR: X-linked juvenile retinoschisis, STGD: Stargardt disease, IZ: interdigitation zone, EZ: ellipsoid zone, ELM: external limiting membrane, ONL: outer nuclear layer, CC: choriocapillaris, Rb: retinoblastoma, JIA: juvenile idiopathic arthritis, AS-OCT: anterior segment optical coherence tomography.

## Data Availability

Not applicable.
